# Causal Relationship Between Gut Microbiota and Female Infertility: A Bidirectional Mendelian Randomization Analysis

**DOI:** 10.5935/1518-0557.20260016

**Published:** 2026

**Authors:** Qiuying Gan, Lidan Liu, Bo Liu, Mujun Li

**Affiliations:** 1 Reproductive Center, Nanning Maternity and Child Health Hospital, Nanning, Guangxi, China; 2 Guangxi Reproductive Medical Center, The First Affiliated Hospital of Guangxi Medical University, Nanning, China; # These authors contributed equally to this work as co-first authors

**Keywords:** gut microbiota, female infertility, Mendelian randomization, causal relationship

## Abstract

**Objective:**

This study aims to investigate the causal relationship between gut microbiota and female infertility using a Mendelian randomization (MR) approach.

**Methods:**

A bidirectional MR analysis was conducted using genome-wide association study (GWAS) data from European populations. Genetic variants (SNPs) linked to 473 bacterial species were used as instrumental variables. Data on female infertility were obtained from the FinnGen study, while gut microbiota-related genetic data were sourced from the NHGRI-EBI GWAS Catalog. Several MR methods, including inverse-variance weighted (IVW) analysis, were employed to assess causal associations.

**Results:**

Seven bacterial taxa, including *Actinomycetales, Bifidobacterium, Bifidobacteriaceae, Actinobacteria, Prevotella sp002933775, GCA-900199385 sp900320755,* and *CAG-841 sp002479075*, were found to have a protective effect against female infertility. Higher levels of these bacterial taxa were associated with a lower risk of infertility. No evidence of reverse causality was found, indicating a unidirectional relationship between gut microbiota and female infertility.

**Conclusion:**

This study supports the concept of a gut-reproductive axis by establishing causal relationships between specific bacterial species and female infertility, suggesting potential avenues for therapeutic interventions.

## INTRODUCTION

The World Health Organization defines infertility as the inability to achieve a clinical pregnancy after 12 months or more of regular, unprotected intercourse (Zegers-Hochschild *et al*., 2006). Globally, infertility affects approximately one in six couples of reproductive age (WHO, 2023), with nearly one in eight seeking medical assistance after being unable to conceive within a year (Anyanwu *et al.*, 2024). In China, the burden of female infertility significantly increased from 1990 to 2019, with 7.06 million additional cases and an average annual growth of 10.10% in the age-standardized prevalence rate (Yu *et al.*, 2023). This rise underscores the need for greater attention to female infertility to reduce the associated health burden.

The gut microbiota plays a pivotal role in various physiological processes, including reproductive function (Xholli *et al.*, 2023). Recently, research into the impact of gut microbiota on female reproductive health has intensified (Wang *et al.*, 2024). Studies have demonstrated that gut microbiota influences female reproductive function through both direct and indirect pathways (Chadchan *et al.*, 2022), impacting reproductive health by regulating hormone levels, the immune system, nutrient metabolism, inflammatory responses, and the microenvironment of the reproductive tract (Xholli *et al.*, 2023). The connection between gut dysbiosis and female infertility is becoming increasingly apparent, with evidence showing that women with infertility often exhibit significant alterations in gut microbiota compared to those with normal fertility (Patel *et al.*, 2022). Nevertheless, direct data linking gut microbiota to female infertility are still limited, and many facets of this relationship remain unexplored.

Traditional observational studies examining the causal relationship between gut microbiota and female infertility are often confounded by behavioral, social, and psychological factors, making it difficult to establish clear causality (Davey Smith & Hemani, 2014). To address these limitations, the current study employs the Mendelian randomization (MR) method. MR leverages genetic variants, such as single nucleotide polymorphisms (SNPs), as instrumental variables (Emdin *et al.*, 2017). These genetic variants are randomly assigned at conception, similar to the randomization in controlled trials, which allows MR to minimize biases from confounding factors and reverse causation, leading to stronger causal inferences (Ference *et al.*, 2021; Nguyen & Mitchell, 2024).

To date, only a few Mendelian randomization studies have explored the relationship between gut microbiota and female infertility. The first study examined the association between 11 bacterial species and female infertility, identifying evidence of horizontal pleiotropy in the inverse variance-weighted (IVW) estimates, which may compromise the reliability of the results (Li *et al.*, 2023). The second study assessed the relationship between 131 bacterial species and female infertility, finding that certain species, such as *Eubacterium ventriosum, Holdemania, Lactococcus, Ruminococcaceae NK4A214*, and *Ruminococcus torques*, had a protective effect, while Faecalibacterium posed a risk factor for infertility. However, the study did not apply p-value corrections, raising concerns about potential false positives (Xi *et al.*, 2023). The third study focused only on bacterial genus-level associations with female infertility (Zhang *et al.*, 2023).

In this study, we aim to evaluate the causal relationship between 473 bacterial species and the risk of female infertility using the MR approach, providing a more robust scientific basis for understanding the etiology of infertility.

## MATERIAL AND METHODS

### Study Design

**Figure 1:**
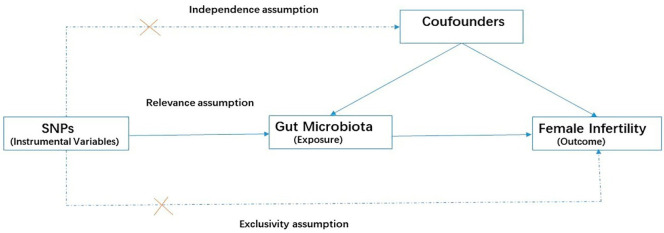
Workflow of the Mendelian Randomization (MR) Design for Investigating the Causal Relationship Between Gut Microbiota and Female Infertility.

A bidirectional Mendelian randomization (MR) analysis was conducted to investigate potential causal links between 473 bacterial species and female infertility. Single nucleotide polymorphisms (SNPs) significantly associated with the exposure variables were selected as instrumental variables (IVs). To ensure robust analysis, SNPs in linkage disequilibrium (LD) or those identified as weak instruments were excluded. For reliable causal inference in MR, three key assumptions must be met: (1) IVs must have a strong association with the exposure; (2) IVs should be free from associations with confounding factors; and (3) IVs must influence the outcome exclusively through the exposure, without a direct effect on the outcome (Figure 1: Workflow of MR design). As the original studies had obtained ethical approval and this analysis utilized publicly available data, no additional ethical approval was required.

### GWAS Summary Data Sources

The summary data on female infertility were obtained from the FinnGen study, a large-scale genomic project, using the R11 release (https://finngen.gitbook.io/) (Kurki *et al.*, 2023). This dataset includes genomic information from 136,188 Finnish individuals, comprising 16,720 infertility cases and 119,468 controls, covering 20,423,066 genetic variants. In this study, female infertility was defined as the inability to conceive after a specific period of unprotected intercourse.

Gut microbiota-related genome-wide association studies (GWAS) data were sourced from the NHGRI-EBI GWAS Catalog, with accessions from GCST90032172 to GCST90032644 (Armet *et al.*, 2022). These genetic variants linked to gut microbiota were identified via GWAS, analyzing 7,979,834 variants in 5,959 participants from the FINRISK 2002 cohort, a population-based study in Finland. The genome-wide analysis revealed 567 distinct SNP-taxon associations with gut microbial taxa (Qin *et al.*, 2022).

### Instrumental Variable Selection

To ensure the validity of instrumental variables (SNPs) for MR analysis, a rigorous selection process was implemented, adhering to MR assumptions. Using the extract instruments function, SNPs strongly associated with the exposure variables were identified, applying a relaxed significance threshold of P < 1×10^-5^ to increase the number of effective IVs (Fu *et al.*, 2024). This threshold was chosen due to the limited number of available IVs when applying a more stringent P < 5×10^-8^ threshold, thereby increasing the sample size.

To ensure the independence of IVs and minimize confounding bias, linkage disequilibrium (LD) analysis with an r^2^<0.001 threshold was conducted, coupled with a clumping strategy over a 10,000 kb window, retaining only the SNP with the lowest *p*-value. SNPs with palindromic sequences or significant allele frequency discrepancies were excluded during the alignment of SNPs across exposure and outcome datasets. The robustness of the selected SNPs as IVs was further evaluated by calculating F-statistics and variance explained (R^2^) to mitigate the risk of bias due to weak instruments (Pierce *et al.*, 2011; Palmer *et al.*, 2012).


$$F=\frac{R^{2}\times (N-2)}{1-R^{2}}$$

$$R^{2}=\frac{2\cdot \beta^{2}\cdot EAF\cdot (1-EAF)}{2\cdot N\cdot EAF\cdot (1-EAF)\cdot SE^{2}}$$

### Statistical Analysis

The Two Sample MR package (version 0.6.6) within R software (version 4.4.1) was used to investigate the bidirectional causal relationship between gut microbiota and female infertility. A range of MR methods were applied, including MR-Egger, weighted median, inverse-variance weighted (IVW), simple mode, and weighted mode. The IVW method was the primary analytical approach (Burgess *et al.*, 2013), with significant IVW results (*p*<0.05) confirming a causal relationship. Cochran’s Q statistic was used to assess heterogeneity among IVs, with *p*-values less than 0.05 indicating significant heterogeneity (Kulinskaya & Dollinger, 2015). Horizontal pleiotropy was evaluated via the MR-Egger intercept, where *p*>0.05 suggested the absence of horizontal pleiotropy. The MR-PRESSO method was employed to detect and correct for outliers, with causal estimates recalculated after excluding identified outliers (Verbanck *et al.*, 2018). A leave-one-out analysis was also performed, systematically excluding each SNP to evaluate its influence on the overall results. For analyses involving large-scale exposure factors, a False Discovery Rate (FDR) correction with a threshold of FDR<0.1 was applied to control for multiple testing and reduce false positives (Benjamini & Yekutieli, 2001).

## RESULTS

**Figure 2:**
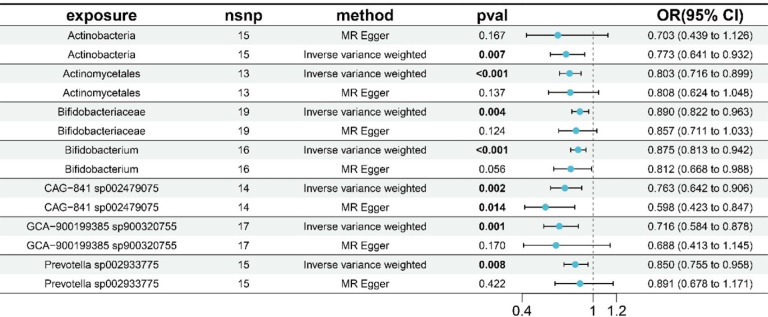
Forest Plot of Causal Associations Between Bacterial Taxa and Female Infertility Using IVW Analysis Based on Mendelian Randomization.

Five distinct Mendelian randomization (MR) methods-IVW, MR-Egger regression, Weighted Median, Weighted Mode, and Simple Mode-were applied using strict instrumental variable selection criteria to explore the potential association between 473 bacterial species and female infertility. The initial IVW analysis (*p*<0.05) identified 32 bacterial species that might be linked to female infertility (Figure 2). However, after applying the False Discovery Rate (FDR) correction (FDR<0.1), ensuring consistent effect direction across all MR methods, and assessing pleiotropy (*p*>0.05), only seven bacterial species were ultimately found to have a causal relationship with female infertility. The reverse analysis did not reveal any significant associations between female infertility and gut microbiota.

### Effects of Gut Microbiota on Female Infertility

According to IVW analysis, seven bacterial taxa were found to have a negative association with female infertility. The following taxa showed significant results:

1. *Actinomycetales* (b=-0.220, OR=0.803, 95% CI=0.716-0.899, *p*=0.0001, FDR=0.0118)
2. *Bifidobacterium* (b=-0.133, OR=0.875, 95% CI=0.813-0.942, *p*=0.0003, FDR=0.0150)
3. *GCA-900199385 sp900320755* (b=-0.335, OR=0.716, 95% CI=0.584-0.878, *p*=0.0013, FDR=0.0351)
4. *CAG-841 sp002479075* (b=-0.271, OR=0.763, 95% CI=0.642-0.906, *p*=0.0021, FDR=0.0463)
5. *Bifidobacteriaceae* (b=-0.117, OR=0.890, 95% CI=0.822-0.963, *p*=0.0039, FDR=0.0591)
6. *Actinobacteria* (b=-0.258, OR=0.773, 95% CI=0.641-0.932, *p*=0.0069, FDR=0.0747)
7. *Prevotella sp002933775* (b=-0.162, OR=0.850, 95% CI=0.755-0.958, *p*=0.0077, FDR=0.0769)

**Figure 3:**
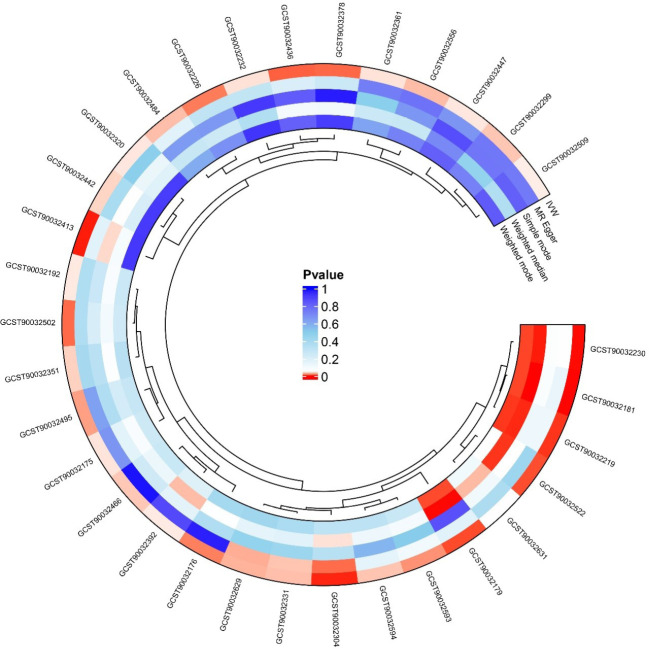
Circular Plot Illustrating Five Mendelian Randomization Analyses of 32 Bacterial Species with Significant Causal Associations to Female Infertility, Highlighting IVW Results (*p*<0.05) in Red.

These results suggest a causal protective effect of higher levels of these bacterial taxa on the risk of female infertility (Figure 3).

**Supplementary Figure 1:**
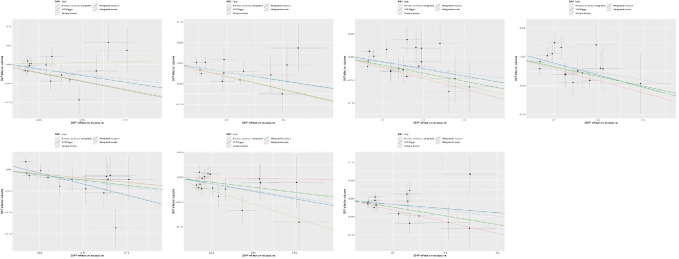
Scatter Plots for Mendelian Randomization Analysis Showing the Relationship Between SNP Effects on Gut Microbiota and Female Infertility.

**Supplementary Figure 2:**
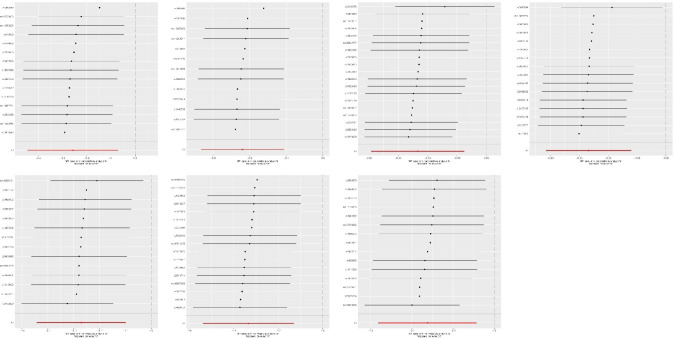
Leave-One-Out Sensitivity Analysis for Mendelian Randomization Assessing the Causal Relationship Between Gut Microbiota and Female Infertility.

There was no significant heterogeneity detected among the instrumental variables using either MR Egger or IVW methods. Furthermore, the MR-Egger intercept did not show any evidence of horizontal pleiotropy, and the MR-PRESSO global test did not detect significant heterogeneity (Table 1). Scatter plots and leave-one-out analysis further validate these findings (Supplementary Figure 1 and 2).

**Table 1:** MR Analysis of exposures and Gut Microbiota: Heterogeneity, Pleiotropy, and MR-PRESSO Results.

Exposure	microbiota	Heterogeneity(pval)		Pleiotropy(pval)	MR-PRESSO(pval)
		MR Egger	IVW		
GCST90032179	Actinobacteria	0.140	0.175	0.675	0.165
GCST90032181	Actinomycetales	0.500	0.586	0.952	0.569
GCST90032219	Bifidobacteriaceae	0.238	0.281	0.665	0.256
GCST90032230	Bifidobacterium	0.407	0.433	0.434	0.417
GCST90032304	CAG-841 sp002479075	0.592	0.467	0.141	0.524
GCST90032413	GCA-900199385 sp900320755	0.515	0.586	0.869	0.605
GCST90032522	Prevotella sp002933775	0.247	0.300	0.715	0.335

### Effects of Female Infertility on Gut Microbiota

Initial inverse-variance weighted (IVW) analysis (*p*<0.05) suggested that female infertility could be linked to 22 bacterial species in the gut microbiota. However, after applying FDR correction (FDR<0.1), no significant effects of female infertility on gut microbiota were observed.

## DISCUSSION

This bidirectional Mendelian randomization (MR) analysis provides a comprehensive evaluation of the causal relationship between 473 bacterial species and female infertility. The results highlight the potential protective effects of specific bacterial taxa, such as *Bifidobacterium, Actinomycetales, Bifidobacteriaceae, Actinobacteria,* and *Prevotella*, in reducing the risk of female infertility. The identification of these seven bacterial species suggests that gut microbiota may play a more significant role in modulating female reproductive health than previously understood (Wang *et al.*, 2024). These findings align with emerging evidence linking gut microbial diversity and composition to various physiological processes, including hormone regulation, immune responses, and metabolic functions, all of which are crucial to reproductive health. The causal associations uncovered in this study not only reinforce the gut-reproductive axis hypothesis but also offer a more precise understanding of how gut microbiota influence female infertility.

Moreover, this study’s rigorous application of the Mendelian randomization framework reduced confounding variables and reverse causation, common challenges in observational studies. The results affirm the robustness of the identified associations, as demonstrated by the lack of significant horizontal pleiotropy and the consistency of results across various MR methods. While previous research suggested correlations between gut dysbiosis and female infertility (Patel *et al.*, 2022), this study strengthens those claims by establishing causal links. However, despite significant findings indicating the influence of gut microbiota on female infertility, the reverse analysis found no evidence that female infertility affects gut microbiota composition. This points to a unidirectional effect, wherein gut microbiota may impact female infertility but not vice versa, contributing a novel insight to our understanding of the gut-reproductive axis.

In contrast to previous studies (Li *et al.*, 2023; Xi *et al.*, 2023; Zhang *et al.*, 2023), this study applied a more comprehensive approach by incorporating False Discovery Rate (FDR) correction to account for multiple comparisons and employing a broader Mendelian randomization analysis across 473 bacterial species. This robust methodology identified seven bacterial taxa, including *Actinomycetales, Bifidobacterium, Bifidobacteriaceae, Actinobacteria, Prevotella sp002933775, GCA-900199385 sp900320755,* and *CAG-841 sp002479075*, which may have a protective effect on female reproductive health. These findings deepen our understanding of the gut-reproductive axis, providing a stronger foundation for future research into how gut microbiota influence female infertility.

In terms of clinical application, this study suggests that modulating gut microbiota could become a novel therapeutic strategy for female infertility. The identification of protective bacterial taxa, such as *Actinomycetales, Bifidobacterium, Bifidobacteriaceae, Actinobacteria, and Prevotella,* opens new opportunities for interventions aimed at improving reproductive health. Potential clinical applications may include probiotics, dietary modifications, or even microbiota transplantation to optimize gut microbiota composition, possibly reducing infertility risks. The mechanisms by which these protective bacterial taxa may influence female fertility are multifaceted. Bifidobacterium and related Actinobacteria are known to produce short-chain fatty acids (SCFAs), particularly acetate, which can modulate systemic inflammation and enhance intestinal barrier integrity, thereby reducing the translocation of pro-inflammatory lipopolysaccharides that may disrupt ovarian function and endometrial receptivity. Additionally, these bacteria play a crucial role in estrogen metabolism through the regulation of β-glucuronidase activity, which influences the enterohepatic circulation of estrogens and maintains optimal hormonal balance essential for ovulation and implantation. Prevotella species have been associated with improved metabolic profiles and reduced oxidative stress, both of which are critical for maintaining reproductive health. Furthermore, these beneficial bacteria may enhance the production of essential nutrients such as folate and B vitamins, which are vital for DNA synthesis and cellular function in reproductive tissues. Personalized gut microbiota-based treatments may complement traditional fertility interventions, offering more targeted and individualized therapeutic strategies.

However, several limitations of this study must be acknowledged. While Mendelian randomization is a powerful method for inferring causality, its effectiveness depends on the quality and completeness of the genetic and microbiome data used. This study relied primarily on data from European populations, which may limit the generalizability of findings to other ethnic groups. Furthermore, the complexity and diversity of the gut microbiome present challenges in fully elucidating the mechanisms underlying the observed associations.

## CONCLUSION

In conclusion, this study provides new insights into the potential causal relationship between gut microbiota and female infertility using Mendelian randomization analysis. The identification of seven bacterial taxa, including *Actinomycetales, Bifidobacterium, Bifidobacteriaceae, Actinobacteria, Prevotella sp002933775, GCA-900199385 sp900320755,* and *CAG-841 sp002479075*, which are inversely associated with the risk of female infertility, highlights the crucial role of gut microbiota in reproductive health. These findings reinforce the gut-reproductive axis hypothesis and suggest that targeted microbial interventions, such as probiotics or dietary modifications, could be explored as promising approaches in future fertility treatments.
